# Molecular Processes of Dodder Haustorium Formation on Host Plant under Low Red/Far Red (R/FR) Irradiation

**DOI:** 10.3390/ijms23147528

**Published:** 2022-07-07

**Authors:** Hangkai Pan, Yi Li, Luxi Chen, Junmin Li

**Affiliations:** 1 School of Life Sciences, Shanghai Normal University, Shanghai 200234, China; hangkaipan@126.com; 2 Zhejiang Provincial Key Laboratory of Plant Evolutionary Ecology and Conservation, Taizhou University, Taizhou 318000, China; yooyyi1@163.com (Y.L.); chenluxi88@163.com (L.C.)

**Keywords:** R/FR ratio, *Cuscuta chinensis*, *Arabidopsis thaliana*, haustorium, plant hormone signal transduction, cell wall degradation, lignin biosynthesis

## Abstract

Low R/FR irradiation can promote dodder haustorium formation on the host plant; however, the mechanisms underlying the process are still unknown. In this study, we compared the transcriptomic data during the formation of haustorium of *Cuscuta chinensis* on host plant *Arabidopsis* *thaliana* under low (R/FR = 0.1) versus high (R/FR = 0.2) R/FR irradiation at 12 h, 24 h and 72 h time points. The results show that low R/FR radiation significantly promoted the entanglement and haustorium formation. Transcriptome analysis showed that during the early stage of haustorium formation, low R/FR radiation significantly up-regulated ARR-A related genes and down-regulated peroxidase related genes compared with high R/FR radiation. Meanwhile, during the middle stage of haustorium formation, low R/FR treatment significantly increased the expression of genes related to pectinesterase (PE), polygalacturonase (PG) and pectin lyase (Pel) production, while, during the late stage of haustorium formation, peroxidase (Prx)-related genes were differentially expressed under different R/FR treatments. Overall, our findings show that a low R/FR ratio promotes the parasitism of *C. chinensis* through plant hormone signal transduction and cell wall degradation pathways. This study provides a basis for the control of parasitic plants.

## 1. Introduction

The genus *Cuscuta* (Convolvulaceace) comprises approximately 200 species of parasitic weeds, widely distributed in temperate and subtropical ecosystems [1]. The species under the genus *Cuscuta* acquires water and nutrients by utilizing a specialized structure called haustorium, which establishes vascular connections with a host plant [2]. *Cuscuta* seedlings are self-sufficient for about 2–3 weeks, after which they need a host to survive. Therefore, early detection and attachment to potential hosts via haustoria are crucial for the survival of *Cuscuta* seedlings [3]. Elucidating the molecular mechanisms underlying the formation of haustorium could enhance our understanding of the interaction between the host and parasitic plants and form a basis for controlling parasitic plants. 

The haustorium formation in *Cuscuta* is induced by physical (light and contact cues) and chemical (flavonoid and volatile organic compounds) factors [4,5,6,7]. Previous studies have shown that the light quality, such as blue, red, and far-red light, can influence the parasitization entanglement and haustorium formation in plants [3,8,9]. *C. planiflora* seedlings tend to grow towards the far-red light side when placed under the irradiation of cold white light and far-red light [10]. Exposure to red light (620–700 nm) inhibited the entanglement and haustorium formation of *Cuscuta* seedlings, while the exposure to far-red light modulated stem entanglement and haustorium formation [11,12,13,14]. Different combinations of red and far-red light have different effects on the growth of *Cuscuta*. For example, brief exposure to red light followed by flashes of far-red light can inhibit entanglement and haustorium formation, while prolonged exposure to red light followed by far-red light can promote entanglement and haustorium formation [15]. Haidar et al. (1999) reported that haustorium induction in *Cuscuta* occurs when the R/FR ratio is between 0.02 and 1.0 (0.1 is optimal) [16]. Benvenuti et al. (2005) found that *C. campestris* tended to grow in the lower R/FR = 0.15 region (compared with R/FR = 0.23), and the difference was significant [17]. Under natural conditions, the host plant absorbs red light for photosynthesis, and the far-red light penetrates the plant, resulting in a decrease in R/FR on the side of the host plant. Subsequently, dodder tends to grow in the direction of low R/FR to find the host plant [8]. However, the molecular and developmental mechanisms underlying the formation of haustorium formation under an optimal R/FR ratio remain unclear.

**Figure 1:**
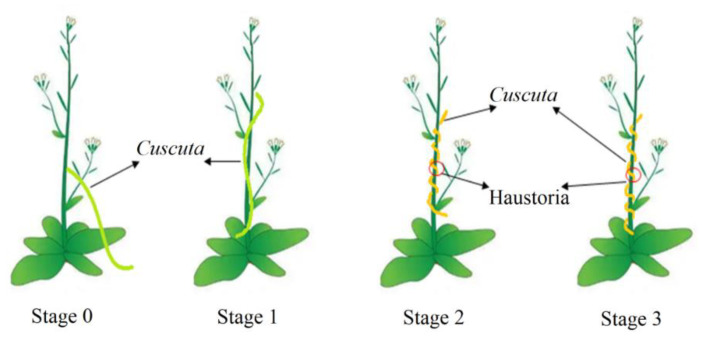
Stages of *Cuscuta* haustoria formation on *Arabidopsis* host Plant. Stage 0: the prehaustorial stage when *Cuscuta* responds to the host; stage 1: the early stage when the haustoria are inducted by the host; stage 2: the middle stage when the haustoria invade the host; stage 3: the later stage when the haustoria mature.

Haustoria formation can be divided into four stages, including the prehaustorial stage (stage 0), the early stage (stage 1), middle stage (stage 2) and later stage (stage 3) (Figure 1). The main occurrence in the prehaustorial stage is the twining response of dodder [3]. The early, middle and later stages refer to haustorium induction, invasion and maturation, respectively [5]. A few studies have reported on the molecular mechanism behind haustoria formation. Li et al. (2010) employed proteomics to identify proteins differentially expressed in dodder seedlings and found that phytochrome, cryptochrome, Ca^2+^/calmodulin and gibberellins (GA) induce the twining response of *Cuscuta australis* in blue light [3]. They also revealed that *C. australis* might contribute to haustorium formation by synthesizing and secreting pectinesterase that degrades the cell wall of host plants. Ranjan et al. used assembled transcriptome to dissect transcriptional dynamics during dodder development and parasitism and identified key gene categories involved in plant parasitism in natural light [18]. According to their findings, Auxin, GA and strigolactone activity increased dodder development and parasitism. Additionally, they observed the upregulation of genes related to stimulus response (such as basic pathogenesis related proteins, heat-shock proteins and drug resistance) and cell wall modification enzymes (proteinspectin lyase, pectin methylesterase and cellulase), suggesting that these genes play a crucial role in haustorial formation. Meanwhile, Rezaei et al. observed that the migration of signals from the host to the parasitic plant up-regulated specific genes (ATP-dependent RNA helicase, glycosylphos-phatidylinositol mannosyltransferase (GPI-MTs), calcium-dependent protein kinase, and heme-binding protein) to overcome plant defense responses, thus promoting the connection between dodder and host plants [19]. However, systematic research on the whole process of haustorium formation is still lacking. In the present study, we examined haustorium formation in parasitic *C. chinensis* in response to host *A. thaliana* under different R/FR ratio (0.1 vs. 0.2) and used transcriptomic data to uncover the potential key genes and transcriptomic factors involved in the four stages of haustorium formation. Our results provide new insights into understanding how the R/FR ratio affects haustorium formation and offer a basis for controlling parasitic plants.

## 2. Results

### 2.1. Effect of R/FR Ratio on Writhing and Haustoria Formation

**Figure 2:**
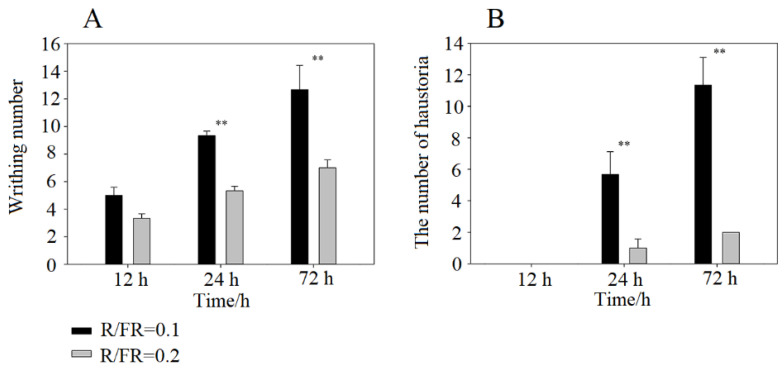
Writhing number (**A**) and haustoria number (**B**) formed by *Cuscuta chinensis* under different R/FR ratios at 12 h, 24 h and 72 h irradiation. ** indicate *p* < 0.01.

At 12 h of irradiation, *C. chinensis* successfully writhed around the stem of *A. thaliana* (Figure 1A). No significant difference in writhing was found between low and high R/FR ratios. Increasing the irradiation time significantly increased the writhing of *C. chinensis* under a low R/FR ratio (*p* < 0.01), but the writhing did not change under a high R/FR ratio (*p* > 0.05) (Figure 2A). The writhing number of *C. chinensis* at a low R/FR ratio was significantly higher than that at high R/FR ratio at 24 or 72 h (Figure 2B).

The haustoria of *C. chinensis* successfully formed on the stem of *A. thaliana*, at 24 h of irradiation (Figure 2B). Increasing the irradiation time significantly increased the haustorium number under a low R/FR ratio (*p* < 0.01), but the haustorium number did not change under a high R/FR ratio (*p* > 0.05) (Figure 2B). The number of haustoria was significantly higher at a low R/FR ratio than at a high R/FR ratio at 24 or 72 h (Figure 2B).

### 2.2. Scanning Electron Microscopy Analysis of Haustoria

**Figure 3:**
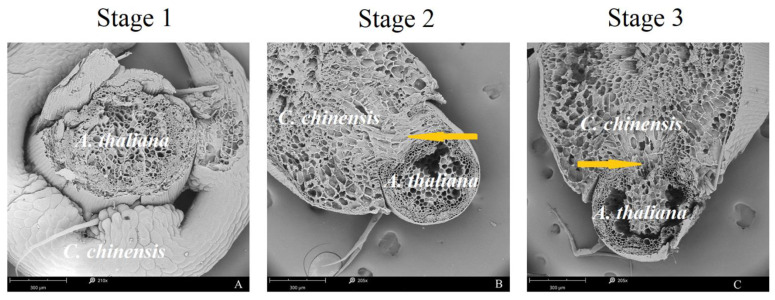
Scanning electron microscope images of haustorial invasion in *Arabidopsis thaliana* at different stages under continuous irradiation at R/FR = 0.1 ratio. (**A**): R/FR = 0.1, 210×, stage 1; (**B**): R/FR = 0.1, 205×, stage 2; (**C**): R/FR = 0.1, 205×, stage 3. The yellow arrow indicates haustorium structure.

Significant changes in the ultramicroscopic structure of haustoria were observed during different stages of haustorium formation under the low R/FR continuous irradiation (Figure 3). In stage 1, epidermal cells of the *C. chinensis* stem in contact with the *A. thaliana* elongated towards the contact surface of the *A. thaliana* epidermis and divided anticlinally to become digitate in form (Figure 3A). In stage 2, the haustorium broke through the cuticle of *A. thaliana* stem and invaded the stem tissue continuously (Figure 3B). In stage 3, the haustorium invaded and connected to the vascular tissue of the *A. thaliana* stem (Figure 3C). A low R/FR ratio promoted the formation of haustorium of *C. chinensis* on host *A. thaliana* (Figure 3).

### 2.3. Overview of Transcriptomic Data

Transcriptome analysis was conducted on the samples from the four stages to further understand the gene expression of dodder at different stages under the different proportions of R/FR continuous irradiation. Transcriptome sequencing was generated at stage 0, stage 1, stage 2, and stage 3 under a low R/FR ratio (L0, L1, L2, and L3) and high R/FR ratio (H0, H1, H2, and H3), in that order (Table S1). After removing the adapter sequence and low-quality sequence, 161.96 GB of clean data were obtained for subsequent analysis. The quality evaluation of transcriptome data showed that the error rate is very low (0.02–0.03%), indicating that we generated high-quality transcriptome data. All the clean reads were assembled using Trinity to generate 294,364 transcripts with an average length of 1490 bp (Figure S1A). A total of 103,882 single genes with an average length of 1134 bp were obtained by clustering; the length of the genes ranged between 301 and 15,800 bp (Figure S1B). For functional annotation of 103,882 single genes, the gene sequence similarity was determined by comparing the data with seven protein databases (the seven databases contain comprehensive gene function information) using BlastX. In total, 57,830 genes (55.66%) were annotated in the NR database, 47,493 genes (45.71%) in the NT database, 40,949 genes (39.41%) in the SwissPort database, 39,774 genes (38.28%) in the PFAM database, 39,772 genes (38.28%) in the GO database, and 20,216 genes (19.46%) in the KO database, while only 14,794 genes (14.24%) were annotated in the KOG database (Figure S1C). Among the results of NR annotation, 35.85% of unigenes were annotated to *C. australis*, 33.6% to *A. thaliana*, 4.6% to *Ipomoea nil*, 2.2% to *Quercus suber*, 1.0% to *Pythium insidiosum*, and 22.9% with other species (Figure S1D).

### 2.4. DEGs in Response to Dodder Parasitism

**Figure 4:**
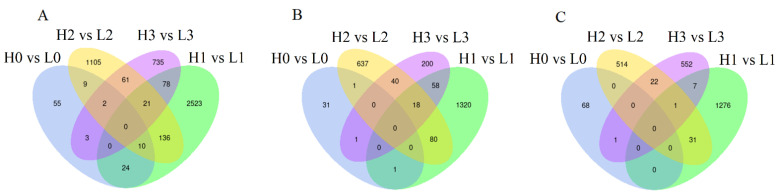
Venn diagram showing the differentially expressed genes (DEGs) in the 4 stages. (**A**) Total DEGs; (**B**) up-regulated DEGs; (**C**) down-regulated DEGs. L0, L1, L2, and L3 indicate the transcriptomic data at stage 0, stage 1, stage 2, and stage 3 under low R/FR ratio (0.1), while H0, H1, H2, and H3 indicate the transcriptomic data at stage 0, stage 1, stage 2, and stage 3 under high R/FR ratio (0.2).

The determination standard of DEGs is | log_2_ (foldchange) | ≥ 1, *p* ≤ 0.05. A total of 103 DEGs were identified in stage 0 (H0 vs. L0) (Figure 4A). Compared with H0, 35 genes were up-regulated (Figure 4B), while 68 genes were down-regulated in L0 (Figure 4C). In stage 1 (H1 vs. L1), 2792 DEGs were identified (Figure 4A). Compared with H1, 1477 genes were up-regulated (Figure 4B), while 1315 genes were down-regulated in L1 (Figure 4C). In stage 2 (H2 vs. L2), 1344 DEGs were identified (Figure 4A). Compared with H2, 776 genes were up-regulated (Figure 4B), while 568 genes were down regulated in L2 (Figure 4C). A total of 900 DEGs were identified in stage 3 (H3 vs. L3) (Figure 4A). Compared with H3, 317 genes were up-regulated (Figure 4B), while 583 genes were down-regulated in L3 (Figure 4C). A total of 24 DEGs were found in stage 0 and 1, 10 DEGs both in stage 0, 1 and 2, and 21 DEGs both in stage 1, 2, and 3 (Figure 4A).

### 2.5. GO and KEGG Enrichment Analysis of DEGs

GO and KEGG enrichment analysis were performed to determine the function of the DEGs. A total of 476 GO terms were enriched in stage 0 (H0 vs. L0), of which 68 were significantly enriched (corrected *p*-value < 0.05; Figure S2A). In stage 1 (H1 vs. L1), 2934 terms were enriched, of which 219 terms were significantly enriched (corrected *p*-value < 0.05; Figure S2B). Meanwhile, 2304 terms were enriched in stage 2 (H2 vs. L2), of which 165 were significantly enriched (corrected *p*-value < 0.05; Figure S2C). In stage 3 (H3 vs. L3), 2103 terms were enriched, of which 211 were significantly enriched (corrected *p*-value < 0.05; Figure S2D).

The DEGs were also subjected to KEGG pathway enrichment analysis. In stage 0 (H0 vs. L0), the DEGs were mainly enriched in plant hormone signal transduction (ko04075, three genes), plant–pathogen interaction (ko04626, one gene), and phenylpropanoid biosynthesis (ko00940, one gene; Figure S3A). In stage 1 (H1 vs. L1), the DEGs were mainly enriched in ribosome (ko03010, 129 genes), plant hormone signal transduction (ko04075, 26 genes), plant–pathogen interaction (ko04626, 12 genes), and phenylpropanoid biosynthesis (ko00940, 8 genes; Figure S3B). In stage 2 (H2 vs. L2), the DEGs were mainly enriched in ribosome (ko03010, 58 genes), plant hormone signal transduction (ko04075, 13 genes) phenylpropanoid biosynthesis (ko00940, seven genes; Figure S3C). In the stage 3 (H3 vs. L3), the DEGs were mainly enriched in ribosome (ko03010, 27 genes), phenylpropanoid biosynthesis (ko00940, 16 genes), plant hormone signal transduction (ko04075, 15 genes), and plant–pathogen interaction (ko04626, six genes; Figure S3D).

### 2.6. Gene Expression Involved in the Prehaustorial and Early Stage of Haustorium Formation

**Figure 5:**
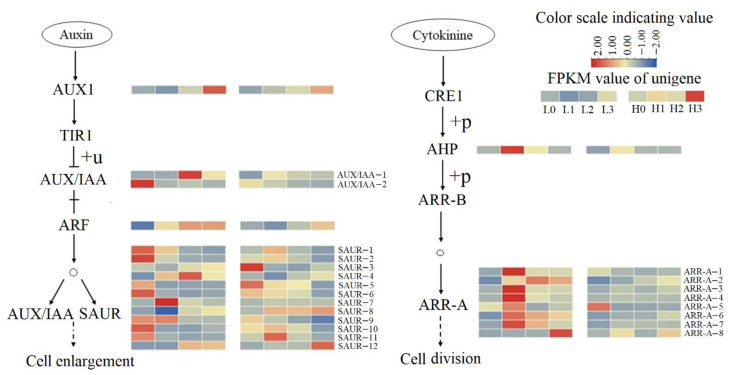
Auxin and cytokinin signaling pathways. FPKM values of unigenes of the enzymes are indicated from blue to red (low to high) across stage 0, stage 1, stage 2, and stage 3 under low R/FR ratio (L0, L1, L2, and L3) and high R/FR ratio (H0, H1, H2 and H3). Dotted lines indicate more than two reaction steps in the biological process. AUX1: auxin influx carrier; TIR1: transport inhibitor response 1; AUX/IAA: auxin/indole-3-acetic acid; ARF: auxin response factor; SAUR: small auxin up-regulated RNA; “†” means ARF dissociates from IAA; “+u” means ubiquitination; CRE1: cytokinin receptor; AHP: *Arabidopsis* histidine-containing phosphotransfer protein; ARR-B: Type-B *Arabidopsis* response regulator; AAR-A: Type-A *Arabidopsis* response regulator; “+p” means phosphorylation. “o” indicates DNA, which was bound by ARF to activate the transcription of AUX/IAA and SAUR, and also bound by ARR-B to activate ARR-A.

Compared with H0, two genes related to small auxin up-regulated RNA (*SAUR-1* and *SAUR-2*) genes were up-regulated, while *SAUR-3* was down-regulated in L0 (Figure 5). 

**Figure 6:**
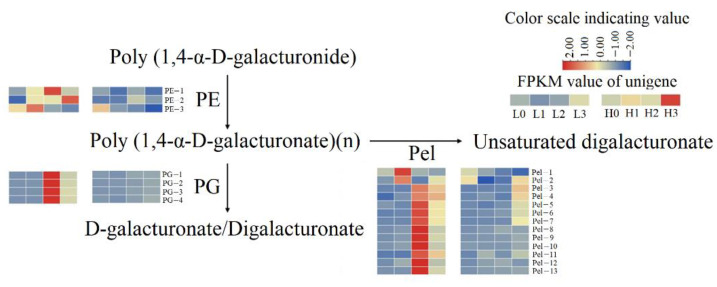
Pectin cleavage pathway. FPKM values of unigenes of the enzymes are indicated from blue to red (low to high) across the stage 0, stage 1, stage 2, and stage 3 under low R/FR ratio (L0, L1, L2, and L3) and high R/FR ratio (H0, H1, H2 and H3). PE: pectinesterase; PG: polygalacturonase; Pel: pectate lyase.

Comparing H1 and L1, seven DEGs were enriched in pentose and glucuronate interconversions. Two genes related to pectin lyase (*Pel-1*, *Pel-2*) and three genes related to pectinesterase (*PE-1*, *PE-2*, *PE-3*) were highly expressed (Figure 6). A total of 18 DEGs were enriched in auxin and cytokinin signal transduction pathways. Among them, one gene related to *Arabidopsis* histidine phosphotransfer (AHP), seven genes related to Type-A *Arabidopsis* response regulator (*ARR-A-1*, *ARR-A-2*, *ARR-A-3*, *ARR-A-4*, ARR-A-5, *ARR-A-6*, *ARR-A-7*), three genes related to SAUR (*SAUR-4*, *SAUR-7*, *SAUR-9*), and one gene related to auxin response factor (ARF) were highly expressed in L1 phase. Five genes related to SAUR (*SAUR-5*, *SAUR-6*, *SAUR-8*, *SAUR-10*, *SAUR-11*) were down-regulated (Figure 5).

### 2.7. Gene Expression Involved in the Middle Stage of Haustorium Formation

**Figure 7:**
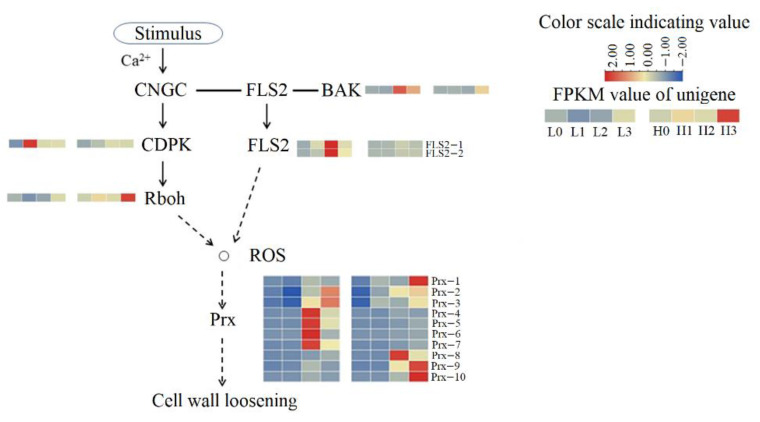
Cell wall loosening process. FPKM value of unigenes of the enzymes are indicated from blue to red (low to high) across the stage 0, stage 1, stage 2, and stage 3 under low R/FR ratio (L0, L1, L2, and L3) and high R/FR ratio (H0, H1, H2 and H3). CNGC: cyclic nucleotide gated channel; FLS2: Flagellin sensitive2; BAK: brassinosteroid-associated kinase; CDPK: calcium-dependent protein kinase; Rboh: respiratory burst oxidase homologs; “o” indicates reactive oxygen species (ROS) resulting from the enrichment of peroxidase (Prx). Dotted lines indicate that there are more than two reaction steps in the biological process.

Compared with H2, 11 genes related to Pel (*Pel-3*, *Pel-4*, *Pel-5*, *Pel-6*, *Pel-7*, *Pel-8*, *Pel-9*, *Pel-10*, *Pel-11*, *Pel-12*, *Pel-13*) and four genes related to polygalacturonase (*PG-1*, *PG-2*, *PG-3*, *PG-4*) were up-regulated in L2 (Figure 6). In addition, *ARR-A-3*, *ARR-A-6*, *ARR-A-7*, *SAUR-12* and *IAA-1* were up-regulated in L2 (Figure 5). In the reactive oxygen species (ROS) generation pathway, genes related to flavonol synthase2 (*FLS2-1*, *FLS2-2*) and brassinosteroid-associated kinase (BAK) are up-regulated in L2. Four genes related to peroxidase (*Prx-4*, *Prx-5*, *Prx-6*, *Prx-7*) were also up-regulated in L2 (Figure 7).

### 2.8. Gene Expression Involved in the Late Stage of Haustorium Formation

**Figure 8:**
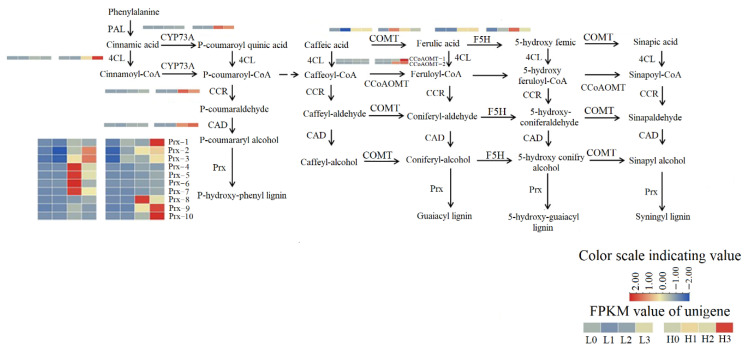
Lignin synthesis pathway. FPKM value of unigenes of the enzymes are indicated from blue to red (low to high) across stage 0, stage 1, stage 2, and stage 3 under low R/FR ratio (L0, L1, L2, and L3) and high R/FR ratio (H0, H1, H2 and H3). PAL: phenylalanine ammonia-lyase; CYP73A: trans-cinnamate 4-monooxygenase; 4CL: 4 coumarate CoA ligase; Prx: peroxidase; CCR: cinnamoyl CoA reductase; CAD: cinnamyl-alcohol dehydrogenase; COMT: caffeic acid 3-O-methyltransferase; CCoAOMT: Caffeoyl-CoA O-methyltransferase; F5H: ferulate-5-hydroxylase.

Compared with H3, most genes related to the phenylpropanoid biosynthesis pathway were down-regulated in L3. These included one gene related to cinnamate 4-hydroxylase (CYP73A), one gene related to 4-coumarate: CoA ligase (4CL), one gene related to cinnamoyl-CoA reductase (CCR), two genes related to Caffeoyl-CoA O-methyltransferase (CCoAOMT), and four genes related to peroxidase (*Prx-1*, *Prx-8*, *Prx-9*, *Prx-10*) (Figure 8).

### 2.9. RT-qPCR Verification

Eight genes closely related to haustorium formation were selected for RT-qPCR. The eight genes exhibited high expression levels and were differentially expressed at four different stages under a low R/FR ratio and high R/FR ratio (Figure S4). These validation results are consistent with the expression trend of sequencing results, indicating that the transcriptome data were reliable.

## 3. Discussion

In this study, a low R/FR ratio increased the writhing and haustorium number of *C. chinensis* on host *A. thaliana*, indicating that a low R/FR ratio can promote haustorium formation of dodder on host plants. Moreover, we identified specific transcripts at the four stages of haustorium formation in *C. chinensis* during low and high F/ FR irradiation and highlighted genes associated with key events in the process. The results showed that low R/FR promotes the parasitism of *C. chinensis* through plant hormone signal transduction (auxin and cytokinin) and the cell wall degradation pathway.

During the prehaustorial and early stage of haustorium formation, *C. chinensis* creates a wound on *A. thaliana*. Haustoria are produced after the dodder has been wrapped around the host plant several times, suggesting that twining into the host plant is a prerequisite for haustorium formation [8]. Dodders twining into the host plant occur through plant tropism (phenomenon of directed growth and deformation in response to stimuli). At the cellular level, deformations occur through anelastic expansions of the cell walls in response to turgor-induced tension [20,21]. The uneven lateral distribution of auxin is widely regarded as a tropism mechanism. Higher levels of auxin are generally associated with faster growth, which induces bending of the entire plant [22,23,24]. Therefore, relaxation of the cell wall and cell elongation are the main processes that occur when dodder wraps around its host. 

Previous studies showed that the haustorial cells of dodder expanded, and the number of haustorial cells increased when the haustoria invaded host plants [25,26]. Auxin and cytokinin can induce haustoria formation under dark conditions [5,27]. In this study, *AUX1*, *AUX*, *SAUR* genes associated with auxin response and *CRE1* and *A-ARR* genes associated with cytokinin response were up-regulated under low R/FR conditions. Moreover, during the early stage of haustorium formation, ARR-A-related genes were significantly up-regulated under low R/FR irradiation relative to high R/FR irradiation. These results suggest that *Cuscuta* growing under a low R/FR ratio (R/FR = 0.1) can increase the production of auxin and cytokinins and potentially enhance cell enlargement and division needed for haustorium formation.

Auxin enrichment can promote the release of ARF from AUX/IAA protein and activate the expression of SAUR genes to facilitate cell enlargement [28]. Cytokinin enrichment can activate ARR-B and ARR-A proteins to promote cell division [29]. Our results indicate that the up-regulated genes related to auxin signal transduction pathways might play essential roles in promoting cell enlargement during the winding process at the back side of *Cuscuta* haustoria, while the up-regulated genes related to cytokinin signal transduction pathways can promote cell division at the haustorial formation site during the early stage of haustorium formation under low R/FR irradiation. 

AUX/IAA and SAUR family proteins are classes of auxin-responsive proteins [30]. Ma and Li (2019) found that *SAUR-9*, *10*, *19*, *20*, *22*, and *23* might be involved in the shade avoidance response, and SAUR proteins could mediate shade-induced cell elongation by regulating cell wall acidification and loosening [31]. In the present study, genes related to AUX/IAA and SAUR family proteins were highly expressed before haustorium formation. For example, the expression of SAUR protein-related genes (*SAUR-1*, *2*, *4*, *7* and *9*) was up-regulated, which facilitated auxin signal transduction and promoted the entangling of *C. chinensis* on *A. thaliana*. However, we also found that the expression of five SAUR genes (*SAUR-5*, *SAUR-6*, *SAUR-8*, *SAUR-10*, *SAUR-11*) was down-regulated in L1 compared to H1. The down-regulation of SAUR family genes might occur only at the opposite side of the haustoria site. In addition, SAUR genes respond to various stimuli, including auxin, light, abscisic acid, and high temperature, and their expression is tissue specific [32]. Thus, the precise function of SAUR genes and related genes in different tissues need to be further analyzed. 

Phytochrome plays a crucial role in the response of the plant to light changes. Many higher plants avoid dense canopy conditions by detecting the changes in the R/FR ratio via phytochrome [33]. In this study, according to gene annotation, phytochrome genes (*PhyA* and *PhyB*) were up-regulated in *Cuscuta* in response to R/FR continuous irradiation. However, *PhyA* and *PhyB* were not differentially expressed in *Cuscuta* under a low R/FR ratio and high R/FR ratio, suggesting that *PhyA* and *PhyB* genes are not sensitive to the changes in the R/FR ratio from 0.1 to 0.2 in this study. A separate study conducted in our lab revealed that *PhyA* and *PhyB* genes were differentially expressed genes in *Cuscuta* under blue and while light (unpublished data). The mechanism underlying the sensitivity of *PhyA* and *PhyB* genes to changes in the R/FR ratio in response to the host plant should be explored in the future.

During the middle stage of haustorium formation, haustorium penetrates the host cell wall and connects to the host’s vascular system, facilitated by auxin and cytokinin production [34]. The prerequisite to a successful infection is overcoming the mechanical barriers of the host plant, mainly the cuticle and the polysaccharide cell walls [21]. Studies have revealed elevated activities of PMEs, PGs, cellulases and peroxidases in haustorial and near-haustorial tissues of dodders [35,36,37,38,39,40]. In this study, certain genes related to PE, PG, and Pel were up-regulated in the middle and late stages of haustorium formation under low R/FR irradiation compared with high R/FR irradiation. Pel catalyzes the eliminative cleavage of de-esterized pectin, a major component of the primary cell walls of many higher plants [41]. PE converts high meth-oxyl pectin into low methoxyl pectin, which is further hydrolyzed to pectate by PG [42]. At low R/FR, the up-regulation of genes related to Pel, PE, and PG potentially leads to the accumulation of the three enzymes. These enzymes loosen the wall of the dodder’s cells and overcome the cuticle and cell wall of the host plant. This facilitates haustorium invasion into the epidermal cells of host plants to reach vascular tissues and establish material exchange with host plants.

During the late stage of haustoria formation, dodder reprograms haustorial cells to vascular cells, connecting the host’s vascular system to its own to enable the uptake of water and nutrients [43,44]. Morphological studies have confirmed the formation of xylem in the haustorium [45,46] and translocation of the xylem tracer from host to parasite [47]. Lignin is vital for the integrity of the thick secondary cell walls produced in xylem vascular tissues, such as tracheids, vessels, and fibers. Lignin is embedded with polysaccharides and confers essential mechanical properties to the cells required for water transport and structural support [48]. Prxs have also been suggested to have an active role in lignin biosynthesis in many plant species [49,50]. Plant cells produce ROS in the presence of Prx, and some precursors of lignin are catalyzed by amino acid residues exposed by peroxidase to polymerize plant lignin [51,52]. Compared with the high R/FR ratio, the up-regulation of Prx-related genes at the late stage of haustorial formation under low R/FR radiation can promote lignin accumulation during haustorium formation, which enhances the formation of xylem cells at the later stage of haustorial differentiation. Continuous exposure to red light can increase Prx activity in plants [53,54]. In this study, certain Prx-related genes were up-regulated under high R/FR irradiation, possibly due to the higher proportion of red light.

Light cues play an important role in dodder host location and attachment [55]. Johnson et al. (2016) found that host location and subsequent attachment by dodder (*C. campestris* and *C. gronovii*) was dramatically reduced under high R/FR ratio environments compared to the control and low R/FR ratio environments. In this study, we found that the twining and haustoria numbers were significantly reduced under low-R/FR-ratio environments than in high-R/FR-ratio environments. These results indicate that manipulation of the R/FR ratio during the early stages of plant growth may significantly affect the management and prevention of dodder infestations in crops [55].

## 4. Materials and Methods

### 4.1. Plant Materials

Seeds of *A. thaliana* were provided by the Laboratory of Zhongnan Yang, Shanghai Normal University, Shanghai, China. Seeds of *C. chinensis* were collected from a farm in Chifeng City, Inner Mongolia Autonomous Region, China. 

### 4.2. Seeds Germination

*A. thaliana* seeds were placed in a 1.5 mL centrifuge tube and stored in a refrigerator at 4 °C for 2–3 days to avoid light vernalization. After vernalization, *A. thaliana* seedings were cultured in a climate chamber. The soil mixture included peat:vermiculite:perlite in a ratio of 2:1:1, the humidity was 70%, and the temperature was 23 °C. 

*C. chinensis* seeds were immersed in 70% ethanol (2 min), 5% sodium hypochlorite (5 min), and concentrated sulfuric acid (15 min), and finally washed with distilled water. The seeds were cultured in soil and placed in a climate chamber. The soil mixture included peat:vermiculite:perlite in a ratio of 2:1:1, the humidity was 70%, and the temperature was 23 °C.

### 4.3. Treatments and Tissues Collection

When *A. thaliana* plants were 10–15 cm in height, one *A. thaliana* seedling was transplanted into one pot. When the *C. chinensis* was 10–15 cm in height (4 days or so after germination), one *C. chinensis* seedling was put near the host *A. thaliana*. Six pots were prepared for the experiment. Three pots were irradiated continuously at a R/FR ratio of 0.1, while the other three pots were irradiated at R/FR ratio of 0.2. Under low R/FR (0.1) irradiation, 1–2 cm stems of *C. chinensis* near the haustoria were cut as the excised part. Under high R/FR (0.2) irradiation, 1–2 cm stems of *C. chinensis* near the writhing location were cut as the excised part. The excised part was cut at 0 h, 12 h, 24 h, and 72 h of irradiation time points for further analysis, representing stage 0, stage 1, stage 2 and stage 3, respectively. A parts of the tissues was fixed for scanning electron microscopy analysis, and the other part of tissues were quickly immersed in liquid nitrogen for transcriptome sequencing.

### 4.4. Scanning Electron Microscopy Analysis of Haustoria

The haustoria tissues were pumped in FAA fixative solution in a vacuum device for 15 min and fixed overnight. Subsequently, gradient dehydration with different concentrations of ethanol was performed as follow: 30% ethanol stood at 4 °C for 5 min; 50% ethanol stood at 4 °C for 10 min; 70% ethanol stood at 4 °C for 10 min; 80%, 90%, and 95% ethanol stood at 23 °C for 15 min each; and finally, 100% ethanol dehydrated twice at 23 °C for 20 min each time. The samples were then put into a critical point dryer for drying dehydration treatment (EM CPD300, Leica Microsystems GmbH, Wetzlar, Germany). After drying, the samples were placed in a copper platform containing conductive adhesive for scanning electron microscopy [55].

### 4.5. Transcriptome Analysis

Total RNA was extracted from the haustoria tissue of *C. chinensis* using a total RNA extractor (Trizol) Extraction Kit (Sangon Biotechnology, Shanghai, China) according to the manufacturer’s instructions. Three biological replicates were set for each group, with a total of 24 samples. RNA integrity was assessed using the RNA Nano 6000 Assay Kit of the Bioanalyzer 2100 system (Agilent Technologies, Santa Clara, CA, USA).

The library was built with total RNA, and m-RNA was enriched by Oligo (dT) magnetic beads. The m-RNA was randomly interrupted in fragmentation buffer. The first cDNA strand was synthesized in the m-MulLV reverse transcriptase system using fragment m-RNA as the template and random oligonucleotide as the primer. The RNA was degraded by RNaseH, and the second cDNA strand was synthesized by dNTPs in the DNA Polymerase I system. cDNA of about 370–420 bp was screened for PCR amplification, and PCR products were purified with AMPure XP Beads to obtain the library.

After the library was constructed, a Qubit2.0 fluorometer was used for initial quantification. The library was diluted to 1.5 ng/uL, and Agilent 2100 BioAnalyzer was used to measure the insert size of the library. RT-qPCR was utilized to accurately quantify the library (effective concentration of library is higher than 2 nM) to ensure that the library was of good quality. After library quality inspection, Illumina sequencing was performed after pooling different libraries according to the requirements of effective concentration and target on-board data amount, and a 150 bp paired end reading was generated.

The image data measured by the Illumina sequencer were converted into sequence data (reads) by CASAVA base recognition, and the file was in FASTA format. Some data, including reads with joints, reads containing N (N indicates that base information cannot be determined), and low-quality reads (reads with Qphred ≤ 20 base number accounting for more than 50% of the entire read length), were removed to ensure the quality and reliability of data analysis. All subsequent analyses were performed using clean data.

Trinity (version 2.0.6) (Trinity Technologies, Irvine, CA, USA) was used for de novo assembly of the clean reads from the obtained samples [56]. The transcripts were compiled, and gene function was annotated based on the following database: Nr (NCBI non-redundant protein sequence), Nt (NCBI non-redundant nucleotide sequence), Pfam (Protein family), KOG/COG (Clusters of Orthologous Groups of proteins), Swiss-Prot (A manually annotated and reviewed protein sequence database), KO (KEGG Ortholog database) and GO (Gene Ontology). The GO functional annotation was obtained by comparing the transcripts with the Swiss-Prot and TrEMBL databases, and transcript Kyoto Encyclopedia of Genes and Genomes (KEGG) annotation information relied on KAAS acquisition.

Differential expression analysis was performed for two conditions/groups using DESeq2 R package (1.20.0). Genes with an adjusted *p*-value < 0.05 and a fold change ≥1 found by DESeq2 were designated as differentially expressed. For cluster analysis, a heat map was drawn based on the analysis results using Tbtools software (1.098696, GitHub, Inc., San Francisco, CA, USA).

### 4.6. RT-qPCR Validation

The pre-extracted RNA was reverse transcribed into cDNA using a two-step reverse transcription-quantitative polymerase chain reaction (RT-qPCR) kit (vazyme Biotechnology Co., Ltd., Nanjing, China) based on hiscript II reverse transcriptase. Primers were designed using primer5 software, and *UBI* served as the internal reference gene for the normalization of gene expression [57]. Eight genes were selected for RT-qPCR validation. Three technical replicates were used for each gene involved in the validation. Three biological replicates were also used for each group of three samples at different developmental stages. The amplification system was constructed using chamq universal SYBR qPCR Master Mix (Vazyme Biotech Co., Ltd., Nanjing, China) and placed in CFX connect (bio rad Laboratories Inc., Hercules, CA, USA) for real-time fluorescence quantitative PCR analysis. The relative expression of genes was measured using the 2^−^^ΔΔCt^ method. The corrplot software package in R (3.6.1) was used for correlation analysis to verify the reliability of transcriptome data. Origin Pro software (version 8.0) was used to draw graphs based on data [58,59].

## 5. Conclusions

In this study, we analyzed the important events and related genes in various processes of haustoria induction under the low R/FR ratio condition. We found that a low R/FR ratio significantly promotes the winding and haustorium formation by affecting different biological processes in *C. chinensis*. The response of SAUR to low R/FR significantly promoted the entanglement of *C. chinensis* on the *A. thaliana* host compared to a high R/FR ratio. During the early stage of haustorium formation, ROS accumulation and the up-regulation of auxin and cytokinin signal transduction-related genes contributed to haustorial initiation. Meanwhile, during the middle and late stages of haustorium formation, a low R/FR ratio mainly induced the genes related to PE, Pel, PG, and Prx and promoted the haustorial invasion of host and the connection between the vascular tissue of *C. chinensis*-*A. thaliana*. Collectively, the findings of this study form a basis for controlling parasitic plants. However, various morphological, biochemical, and physiological changes in *Cuscuta* in response to host plants under different R/FR ratios should be investigated. Furthermore, transcriptomic data should be further analyzed to reveal the underlying molecular processes.

## Data Availability

RNA-seq data of *Cuscuta chinensis* were deposited in the NCBI SRA database (PRJNA856163).

## Supplementary Materials

The following supporting information can be downloaded at: https://www.mdpi.com/article/10.3390/ijms23147528/s1.

## Abbreviations

SAUR	small auxin up-regulated RNA
AUX1	auxin influx carrier
ARR-A	Type-A *Arabidopsis* response regulator
ARR-B	Type-B *Arabidopsis* response regulator
TIR1	transport inhibitor response 1
AHP	*Arabidopsis* histidine-containing phosphotransfer peotein
ARF	auxin response factor
PE	pectinesterase
Pel	pectate lyase
PG	polygalacturonase
Prx	peroxidase
CNGC	cyclic nucleotide gated channel
Rboh	respiratory burst oxidase
ROS	reactive oxygen species
FLS2	Flagellin sensitive2
CDPK	calcium-dependent protein kinase
PAL	phenylalanine ammonia-lyase
CYP73A	cinnamate 4-hydroxylase
4CL	4 coumarate CoA ligase
CCR	cinnamoyl CoA reductase
CRE	cytokinin receptor
BAK	brassinosteroid-associated kinase
CAD	cinnamyl-alcohol dehydrogenase
F5H	ferulate-5-hydroxylase
CCoAOMT	Caffeoyl-CoA O-methyltransferase
COMT	caffeic acid 3-O-methyltransferase
