# Supplementary figures and images for: Molecular Processes of Dodder Haustorium Formation on Host Plant under Low Red/Far Red (R/FR) Irradiation
Source: Int J Mol Sci. 2022 Jul 7;23(14):7528. doi: 10.3390/ijms23147528 (PMC9322645; doi:10.3390/ijms23147528)

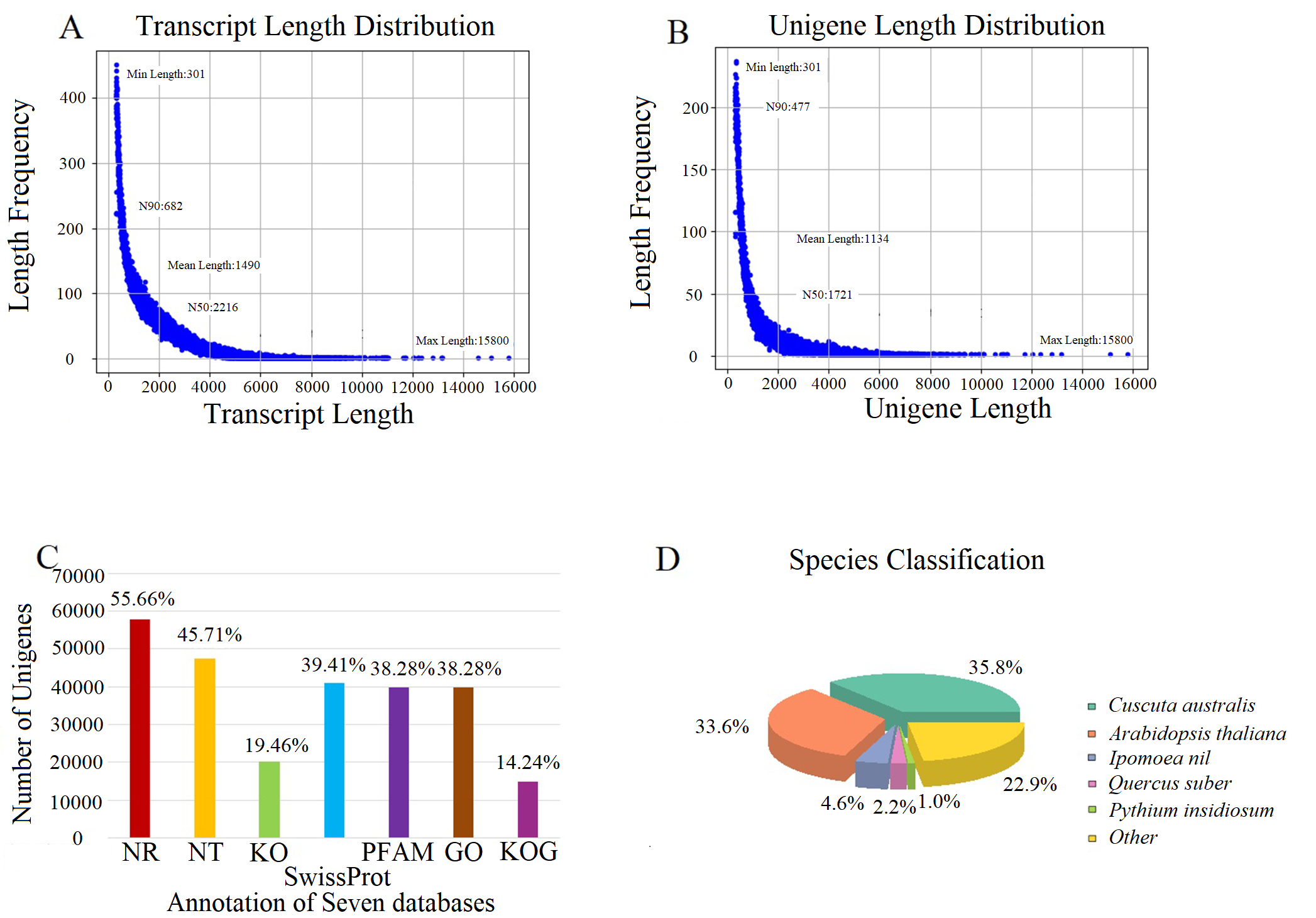

Supplement: Supplementary file 1 [file ijms-23-07528-s001.zip › ijms-1771005-supplementary/Figure S1.tif]

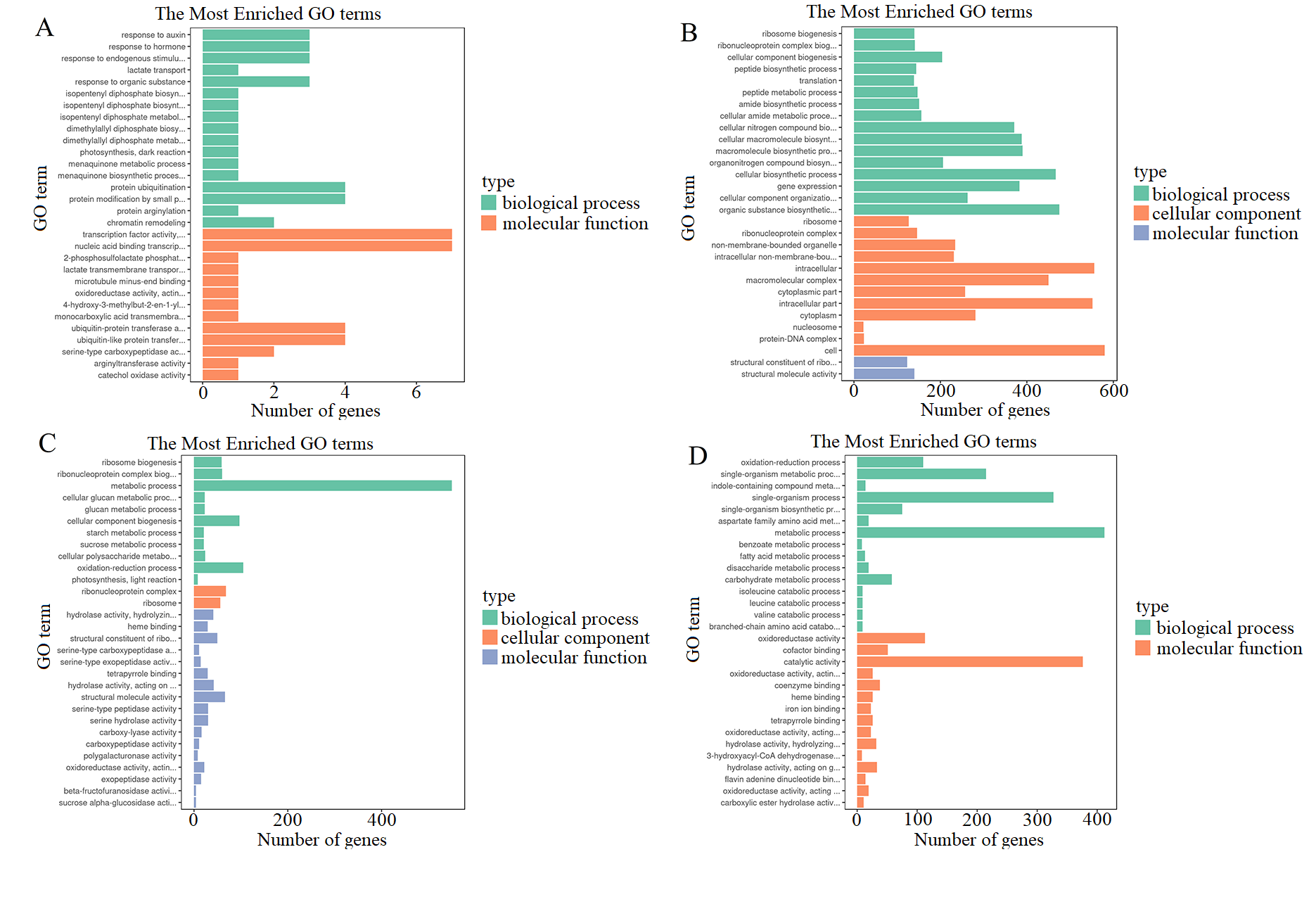

Supplement: Supplementary file 1 [file ijms-23-07528-s001.zip › ijms-1771005-supplementary/Figure S2.tif]

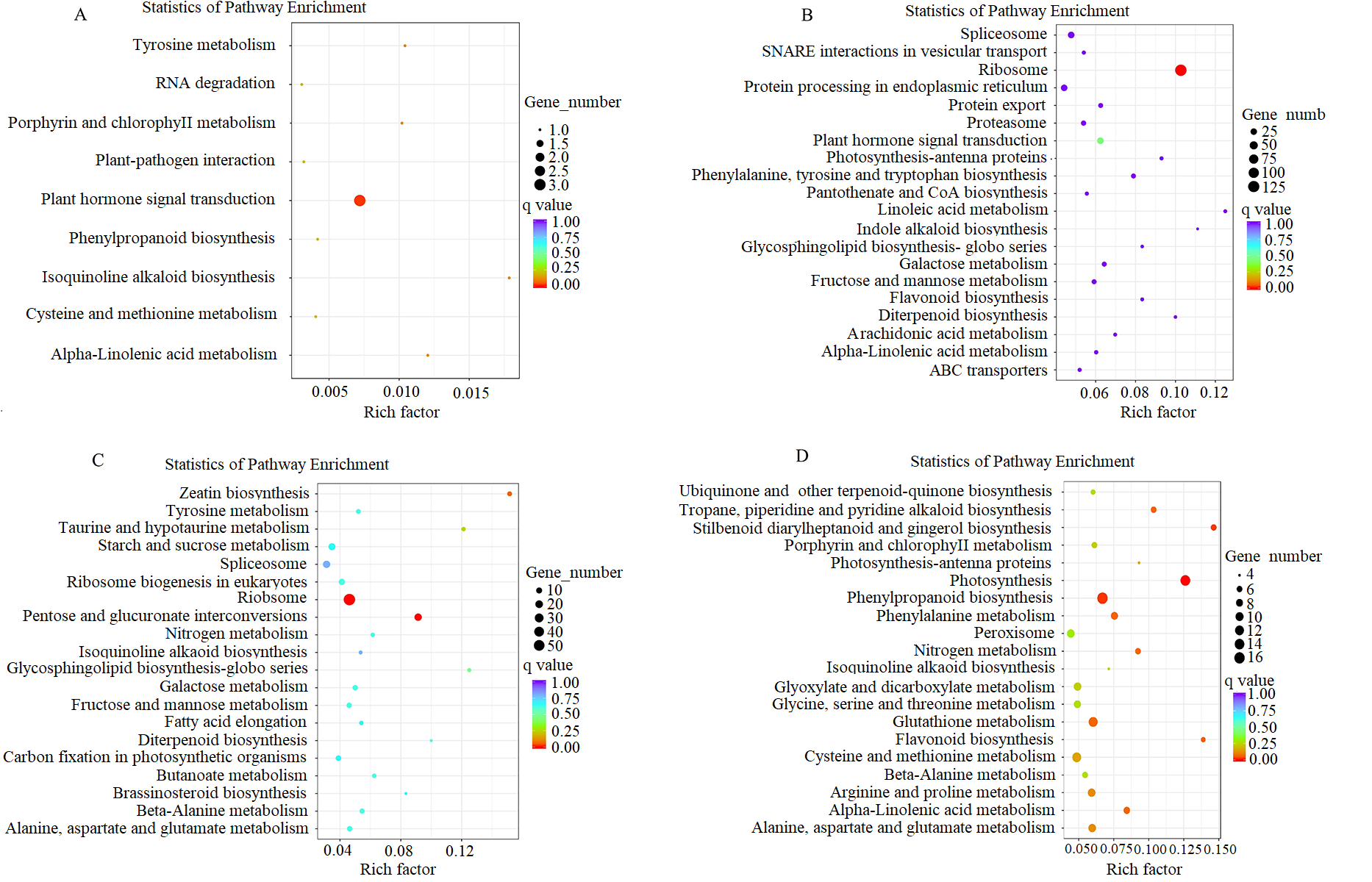

Supplement: Supplementary file 1 [file ijms-23-07528-s001.zip › ijms-1771005-supplementary/Figure S3.tif]

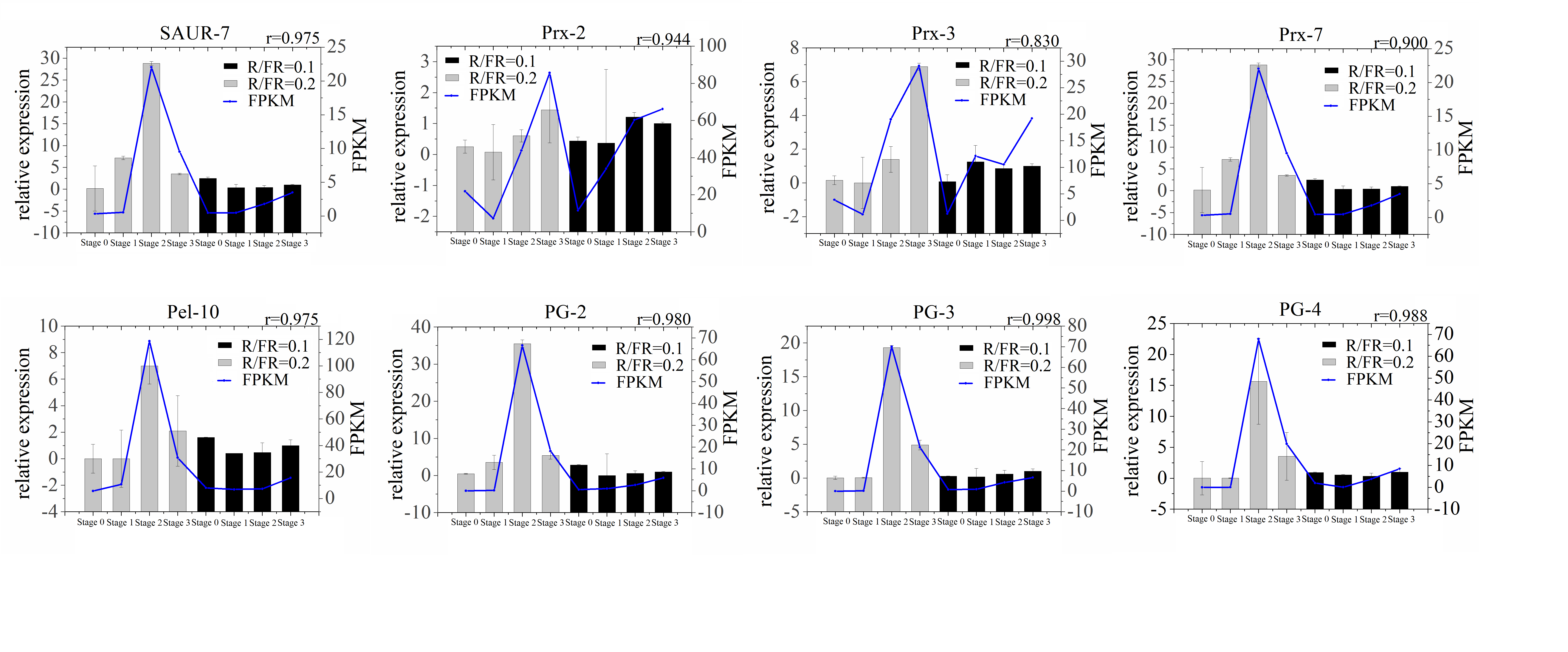

Supplement: Supplementary file 1 [file ijms-23-07528-s001.zip › ijms-1771005-supplementary/Figure S4.tif]
